# Interrelated effects of mycorrhiza and free-living nitrogen fixers cascade up to aboveground herbivores

**DOI:** 10.1002/ece3.1654

**Published:** 2015-08-18

**Authors:** Botir Khaitov, José David Patiño-Ruiz, Tatiana Pina, Peter Schausberger

**Affiliations:** 1 Group of Arthropod Ecology and Behavior, Department of Crop Sciences, University of Natural Resources and Life SciencesPeter Jordanstrasse 82, 1190, Vienna, Austria; 2 Division of Legume Crops, Department of Plant Sciences, Tashkent State Agrarian UniversityUniversitetskaya street 2a, 370, Tashkent, Uzbekistan; 3 Departament de Ciències Agràries i del Medi Natural, Unitat Associada d'Entomologia UJI/IVIA, Universitat Jaume ICampus del Riu Sec, 12071, Castelló de la Plana, Spain

**Keywords:** Aboveground–belowground interactions, arbuscular mycorrhiza, fitness, multitrophic interactions, nitrogen fixation, spider mites

## Abstract

Aboveground plant performance is strongly influenced by belowground microorganisms, some of which are pathogenic and have negative effects, while others, such as nitrogen-fixing bacteria and arbuscular mycorrhizal fungi, usually have positive effects. Recent research revealed that belowground interactions between plants and functionally distinct groups of microorganisms cascade up to aboveground plant associates such as herbivores and their natural enemies. However, while functionally distinct belowground microorganisms commonly co-occur in the rhizosphere, their combined effects, and relative contributions, respectively, on performance of aboveground plant-associated organisms are virtually unexplored. Here, we scrutinized and disentangled the effects of free-living nitrogen-fixing (diazotrophic) bacteria *Azotobacter chroococcum* (DB) and arbuscular mycorrhizal fungi *Glomus mosseae* (AMF) on host plant choice and reproduction of the herbivorous two-spotted spider mite *Tetranychus urticae* on common bean plants *Phaseolus vulgaris*. Additionally, we assessed plant growth, and AMF and DB occurrence and density as affected by each other. Both AMF alone and DB alone increased spider mite reproduction to similar levels, as compared to the control, and exerted additive effects under co-occurrence. These effects were similarly apparent in host plant choice, that is, the mites preferred leaves from plants with both AMF and DB to plants with AMF or DB to plants grown without AMF and DB. DB, which also act as AMF helper bacteria, enhanced root colonization by AMF, whereas AMF did not affect DB abundance. AMF but not DB increased growth of reproductive plant tissue and seed production, respectively. Both AMF and DB increased the biomass of vegetative aboveground plant tissue. Our study breaks new ground in multitrophic belowground–aboveground research by providing first insights into the fitness implications of plant-mediated interactions between interrelated belowground fungi–bacteria and aboveground herbivores.

## Introduction

Plants are the prime links between the below- and aboveground spheres, mediating interactions between below- and aboveground living organisms that do not directly interact with each other. Based on intensive research during the past two decades, it is now generally acknowledged that below- and aboveground plant-associated processes are mutually dependent (Van der Putten et al. 2001; Bezemer and van Dam 2005; Rasmann and Turlings 2007; Erb et al. 2008; Koricheva et al. 2009; Heil 2011; van Dam and Heil 2011; Schausberger et al. 2012). Among others, aboveground plant performance is strongly influenced by belowground microorganisms, which may be either pathogens or mutualists, which in turn affects herbivorous organisms inhabiting and feeding on green plant parts (Van der Putten et al. 2001; Bezemer and van Dam 2005; Van Dam and Heil 2011). Cases in point are plants living belowground in concurrent, usually mutualistic, symbiosis with functionally distinct microorganisms such as nodulating rhizobial bacteria (RB), and/or free-living nitrogen fixers, such as diazotrophic bacteria (DB), and/or plant growth promoting rhizobacteria, such as *Pseudomonas* sp., and/or arbuscular mycorrhizal fungi (AMF) (Pineda et al. 2010, 2013). Concurrent associations with AMF and nitrogen fixers (RB or DB) commonly act additively or synergistically on nutrient uptake by the plants (e.g. El-Shanshoury et al. 1989; Ibijbijen et al. 1996; Barea et al. 2005; Artursson et al. 2006; Sabannavar and Lakshman 2011). However, under certain circumstances, also competition for nutrients, space, or other resources between AMF and nitrogen fixers may occur, resulting in subtractive effects as compared to the predicted sum of each symbiont's effect (Scheublin and van der Heijden 2006).

Recent research provided insights into how belowground interactions between plants and one functional type of microorganism may cascade up to aboveground herbivores (for plant growth promoting rhizobacteria: Tomczyk 2006; Saravanakumar et al. 2008; for AMF: Hoffmann et al. 2009, 2011a,b; for RB: Kempel et al. 2009; Thamer et al. 2011; Dean et al. 2014) and their natural enemies (e.g. Gange et al. 2003; Schausberger et al. 2012; Pineda et al. 2013). Also combined effects of different strains of a given species of microorganism (e.g. Saravanakumar et al. 2008; Roger et al. 2013) or of different, but functionally similar, species of AMF (Gange et al. 2003) as well as microorganism community effects (Hol et al. 2010) have been looked at. However, functionally distinct belowground microorganisms commonly co-occur, but their combined effects and relative contributions, respectively, on aboveground herbivores and their natural enemies are virtually unexplored (Pangesti et al. 2013). Comparing bottom-up effects of belowground plant associations with AMF, nitrogen fixers and both in combination on arthropods living on aboveground plant parts are an important ecological and evolutionary issue in multitrophic research. This issue is also relevant for applied ecology, for example, in agriculture, forestry, or ecosystem restoration, because commercially available, functionally distinct microorganisms are often jointly used for promoting plant growth and health (Sprent and Sprent 1990; Mrkovacki and Milic 2001; Wu et al. 2005; Rathi et al. 2014).

**Figure 1:**
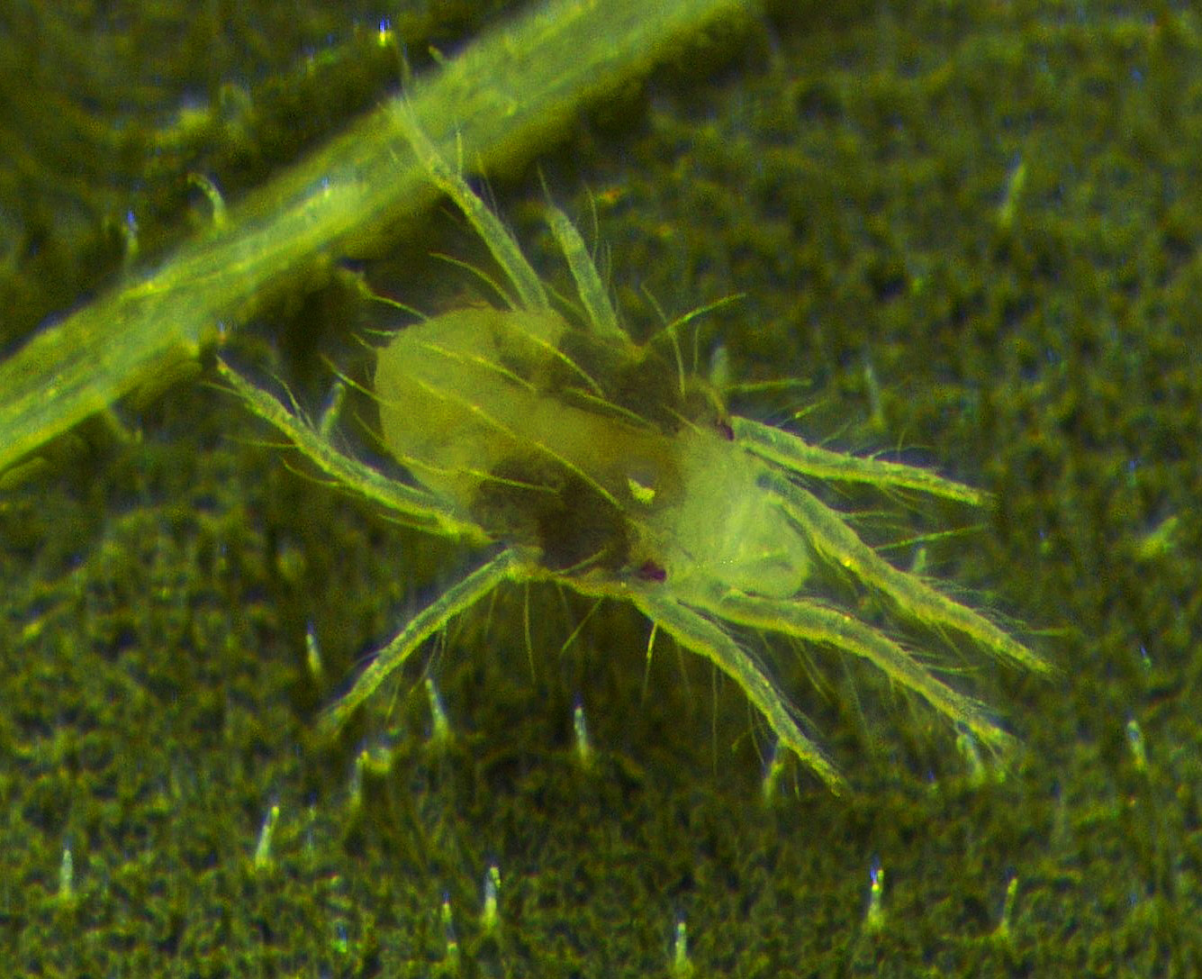
Adult two-spotted spider mite female, *Tetranychus urticae*, on bean leaf, *Phaseolus vulgaris*.

We addressed this novel belowground–aboveground issue in a multitrophic system consisting of common bean plants *Phaseolus vulgaris* L., two belowground microorganisms, the arbuscular mycorrhizal fungus *Glomus mosseae* Nicol. and Gerd. and free-living nitrogen-fixing bacteria *Azotobacter chroococcum* Beijerinck, and an aboveground herbivore, the two-spotted spider mite *Tetranychus urticae* Koch (Fig.1). This belowground–aboveground system is, for its easy experimental accessibility and, except *A. chroococcum*, relatively well understood (Hoffmann et al. 2009, 2011a,b; Schausberger et al. 2012; Patiño-Ruiz and Schausberger 2014), including the effects of nodulating rhizobia (Katayama et al. 2010)*. Tetranychus urticae* is a globally distributed, polyphagous herbivore with >1000 recorded host plant species (e.g. Bolland et al. 1998). The spider mites feed on their host plants by piercing the parenchyma cells and sucking out the cell contents. Their behavioral and life history performance is enhanced by the AMF *G. mosseae* (e.g. Hoffmann et al. 2009, 2011a,b; Patiño-Ruiz and Schausberger 2014). *Glomus mosseae* is a widespread facultative symbiont of vascular plants (Giovannetti et al. 1993). In general, the AMF hyphae penetrate the roots and grow intracellularly, penetrating individual cells and inside forming arbuscules for exchange of resources with the plant (Allen 1996). AMF may affect plant growth and health by changing mineral nutrition, especially phosphorous (P) uptake (Smith and Read 1997; Clark and Zeto 2000), and consequently influences resistance and tolerance to biotic (Trotta et al. 1996; Azcon-Aguilar et al. 2002; Schausberger et al. 2012) and/or abiotic stressors (Turnau and Haselwandter 2002). *Azotobacter chroococcum* is an aerobic free-living soil bacterium, playing an important role for natural nitrogen (N) availability. *Azotobacter* sp. and other DB bind atmospheric N and release N in the form of ammonium ions (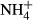), thereby making N accessible to plants (e.g. Mrkovacki and Milic 2001). *Azotobacter* sp. are widely distributed in natural and agricultural soils of temperate regions and more abundant in the rhizosphere of plants, including of legumes, than in soils unassociated with plants (Kole et al. 1988; Rodelas et al. 1999; Mrkovacki and Milic 2001). *Azotobacter* sp. may also function as mycorrhiza helper bacteria (MHB) by enhancing the environmental conditions for AMF occurrence and establishment, for example, by producing growth factors and/or inhibition of competitors and antagonists and/or improving soil conduciveness (e.g. Garbaye 1994; Frey-Klett et al. 2007). The separate effects of AMF and RB on performance of aboveground herbivores are highly species and context dependent, and range from negative over neutral to positive, depending on whether the nutritional value or defensive system of the host plant is more strongly affected (Gehring and Whitham 2002; Kempel et al. 2009; Pineda et al. 2010; Dean et al. 2009, 2014). In contrast, the effects of free-living nitrogen fixers (DB) and the interrelated effects of DB and AMF, which commonly co-occur (Frey-Klett et al. 2007), on aboveground herbivore performance are unknown.

Using the multitrophic system of *P. vulgaris*, *G. mosseae* (AMF), *A. chroococcum* (DB), and *T. urticae*, here we examined (1) how the interactions between AMF and DB affect the host plant choice and life history of the spider mites feeding on aboveground plant parts, (2) how the interactions between AMF and DB affect vegetative and reproductive plant growth, and (3) how AMF and DB affect each other's occurrence, establishment, and growth in the rhizosphere. We conducted two experiments. In the first experiment, we assessed survival and reproduction of the spider mites feeding on leaves of bean plants belowground associated with either *G. mosseae* alone (AMF+/DB−), or *A. chroococcum* alone (AMF−/DB+), or both in combination (AMF+/DB+) or neither of the two microorganisms (AMF−/DB−). Additionally, we quantified vegetative and reproductive plant growth, as affected by the four rhizosphere treatments, and AMF and DB occurrence. In the second experiment, we evaluated host plant preference of adult spider mite females given a choice between four leaves, one from each treatment AMF+/DB−, AMF−/DB+, AMF+/DB+, and AMF−/DB−.

## Materials and Methods

### Plant growing and plant growth measurements

To obtain plants and leaves for experiments, seeds of *P. vulgaris* (var. Taylor's Horticultural) were surface-sterilized with a solution of 75 mL chloride + 25 mL water for 2–3 min, rinsed thoroughly with distilled water and then put in perlite, previously autoclaved for 20 min at 121°C, inside 0.5 L pots for pregermination (20 seeds/pot). Pots and saucers were disinfected with 75% ethanol before use. After 8–10 days, the seedlings, then having the cotyledons and primary leaves, were transplanted in groups of three to 1 L pots filled with a 1 : 1 : 1 silicate sand/expanded clay/soil substrate mixture, previously autoclaved for 20 min at 121°C, and inoculated with either AMF, or DB, or both AMF and DB, or left uninoculated. About 3 weeks after transplanting and inoculation, the plants were ready for use in experiments. Plants were grown under standardized conditions in a walk-in environmental chamber (60 ± 5% RH, 16:8 h L:D, and 23:18°C L:D). The relative placement of pots within the environmental chamber was changed every 2 days to exclude any inadvertent positioning effects. The plants were watered in 2–3 days intervals with ∼80–100 mL per pot of a reduced N and P fertilizer solution (22 mL/L water). The ingredients of the fertilizer solution were K_2_(SO_4_) 0.256 g/L, Mg SO_4_ 0.136 g/L, Fe_6_H_5_O_7_ × 3H_2_O 50.0 g/L, Na_2_Bo_4_O_7_ × 4H_2_O 1.3 g/L, MnSO_4_ × 4H_2_O 1.5 g/L, ZnSO_4_ × 7H_2_O 0.6 g/L, CuSO_4_ × 5H_2_O 0.45 g/L, Al_2_(SO_4_)_3_ 0.028 g/L, NiSO_4_ × 7H_2_O 0.028 g/L, Co(NO_3_)_2_ × 6H_2_O 0.028 g/L, TiO_2_ 0.028 g/L, LiCi_2_ 0.014 g/L, SnCi_2_ 0.014 g/L, KJ 0.014 g/L, KBr 0.014 g/L, and MoO_3_ 0.014 g/L.

After finishing the spider mite experiments and clipping off leaves for experimental use, respectively, which happened ∼6 weeks after transplanting, all remaining aboveground plant material and the roots were removed from the pots for further analyses. The fresh weights of pods, shoots, and roots were measured immediately after removing the plants from the pots, and cleaning the roots, respectively. During cleaning the roots, we also verified the absence of nodules, possibly emanating from inadvertent soil contamination by RB. Subsequently, the pods, shoots, and roots were dried at room temperature (25 ± 2°C and 30–50% RH) for 14 days, and their dry weights measured.

### DB and AMF inoculation and quantification

To inoculate the growing substrate and roots, respectively, with DB, we used a reduced DB solution, prepared by dissolving 2.5 mL of pure Azotovit® (obtained from Agrotrader Agrarhandel, Austria) in 1 L of tap water. Azotovit® consists of pure water containing *Azotobacter chroococcum* at a bacterial density of 5 × 10^9^/mL. For the AMF**−**/DB+ and AMF+/DB+ treatments, during plant transplanting the roots were dipped in the DB solution and 40 mL of the solution was poured into each pot. After 2 weeks, another 40 mL of DB solution was added to each pot.

To estimate the numbers of colony-forming units (cfu) of *A. chroococcum* in the planting substrate, we used an *Azotobacter* sp. specific culturing medium (M372-500G *Azotobacter* Mannitol Agar; HiMedia Laboratories, India). A total of 41.4 g *Azotobacter* sp. medium (containing soil extract 5 g, mannitol 20 g, K_2_HPO_4_ 1 g, MgSO_4_ 0.2 g, NaCl 0.2 g, FeSO_4_ traces, agar 15 g) was suspended in 1 L distilled water, according to the manufacturer's specifications, heated to boiling to completely dissolve the medium, sterilized by autoclaving at 121°C for 15 min, and then poured into sterile Petri dishes for solidification. The plate count technique was used for determining the presence of *A. chroococcum* and estimating its density: 1 g soil substrate was randomly sampled from each pot and diluted to 10^−5^ in distilled water. 1 ml of diluted soil sample was spread on the surface of the agar plates using a sterile glass spreader. All used devices such as tubes, dishes, and bottles were previously autoclaved for 20 min at 121°C to prevent any contamination. After 72 h incubation at 28°C, *A. chroococcum* had formed large, moist colonies, which were counted. All colonies turned dark brown after 5–7 days of incubation indicating *A. chroococcum* identity (Banerjee et al. 2014).

To inoculate the plants with AMF, we used the *Glomus mosseae* inoculum BEG 12 (http://www.kent.ac.uk/bio/beg) (see Hoffmann et al. 2009). For treatments AMF+/DB+ and AMF+/DB−, 5 g of *G. mosseae* inoculum was added to each pot containing three plants. After the experiments, the roots of all plants were checked for mycorrhizal colonization, which allowed assigning a specific fungal colonization level to each pot used in experiments. Plants were removed from pots and the substrate rinsed off the roots with cold tap water. To assess the root length colonized (RLC) by AMF, the roots were cleared by boiling for 10 min in 10% KOH and stained by boiling for 5 min in a 5% black ink (Sheaffer, Ft. Madison, Iowa), household vinegar (equal to 5% acetic acid) solution (Vierheilig et al. 1998). The RLC was estimated according to Newman (1966) and the modified gridline intersect method (Giovannetti and Mosse 1980).

### Spider mite rearing and experiments

Two-spotted spider mites, *T. urticae* (Fig.1), used in experiments derived from a population reared on whole bean plants *P. vulgaris* at room temperature. Plants used for maintaining the stock population were grown in a sand/clay mixture that did neither contain AMF nor DB. To obtain mated spider mite females for experiments, spider mite nymphs were withdrawn from the stock population and placed in groups of 20–30 individuals on leaf arenas from either AMF−/DB−, AMF+/DB−, AMF−/DB+, or AMF+/DB+ plants until reaching adulthood. Arenas consisted of squares (∼2.5 × 2.5 cm) on detached clean trifoliate leaflets placed upside down on moist filter paper covering a water-soaked foam cube (7 × 7 × 5 cm) resting in a plastic box (10 × 10 × 6 cm) half-filled with water. Each leaf arena was delimited by strips of moist tissue paper to prevent mite escaping. The developmental progress of the spider mites was monitored once per day until the mites had reached adulthood and females were mated, respectively (Hoffmann et al. 2009). To assess oviposition, as influenced by AMF and/or DB, the females were singly transferred from the leaflets, on which they matured, to experimental leaf arenas from either AMF−/DB−, AMF+/DB−, AMF−/DB+, or AMF+/DB+ plants (prepared as described above; females maturing on a given treatment were placed on a leaf from the same treatment) and their survival, activity (moving/stationary), and number of laid eggs assessed once per day for five consecutive days. This experiment was carried out in two series with six replicates per treatment per series, hence performed at two plant ages (3–4 and 5–6 weeks after transplanting and inoculation).

To assess spider mite host plant choice, four leaflets, one of each treatment (AMF−/DB, AMF+/DB−, AMF−/DB+, and AMF+/DB+), were arranged in the shape of a cross on moist filter paper covering a water-soaked foam (15 × 15 × 4 cm) resting in a plastic box (20 × 20 × 6 cm) half-filled with water, with all four leaf tips pointing to, and touching each other, in the center. The relative position and sequence of the four leaflets was random and switched between replicates. To enable free movement of the mites, the four leaflets were smoothly connected by a wax bridge. The wax bridge was created by dripping hot wax from a nonfragrant candle onto the touching zone of the leaf tips (Hoffmann et al. 2009). After cooling and solidification of the wax, four mated spider mite females, one from each treatment (AMF−/DB−, AMF+/DB−, AMF−/DB+, and AMF+/DB+), were released in the middle of the bridge and their residence observed every 30 min for the first 3 h and then again after 24 h. After 24 h, the number of eggs present on each leaflet was recorded. Before release, the four females were marked with different tiny water-color dots on their dorsal sides to make them discernible. The choice experiment was replicated 22 times; every choice unit and every female were used only once.

The leaf arenas used for raising the mites to adulthood before the experiments and the experimental no-choice and choice units were stored in an environmental chamber at 25 ± 1°C, 60 ± 5% RH and 16:8 h L:D.

### Statistical analyses

All statistical analyses were carried out using IBM SPSS 21 (IBM Corp. Armonk, NY). In the spider mite no-choice experiment, we used separate generalized estimating equations (GEE) to analyze the influence of DB and AMF presence/absence and plant age on daily egg production (normal distribution, identity link) of the spider mite females over 5 days, aggregated activity (moving/stationary; binomial distribution with logit link), and mortality (yes/no; binomial distribution with logit link). Plant age and observation day were auto-correlated (AR1) and used as inner subject variables. In the spider mite choice experiment, we used GEEs to analyze the residence (lumped data of seven observations; Poisson distribution, log link) and oviposition (Poisson distribution, log link) preference of the mites for the leaflets from AMF−/DB−, AMF+/DB−, AMF−/DB+, or AMF+/DB+ plants (exchangeable correlation structure between the four leaflets of each replicate). Post hoc least significance difference (Sidak and LSD) tests were used to compare treatment pairs. The effects of DB and AMF presence/absence on the fresh and dry weights (exchangeable correlation structure) of pods, shoots, and roots were analyzed by separate GEEs (normal distribution, identity link). RLC was compared between AMF plants with and without DB, and DB density (bacteria counts) was compared between DB plants with and without AMF, respectively, by generalized linear models (normal distribution, identity link).

## Results

**Figure 2:**
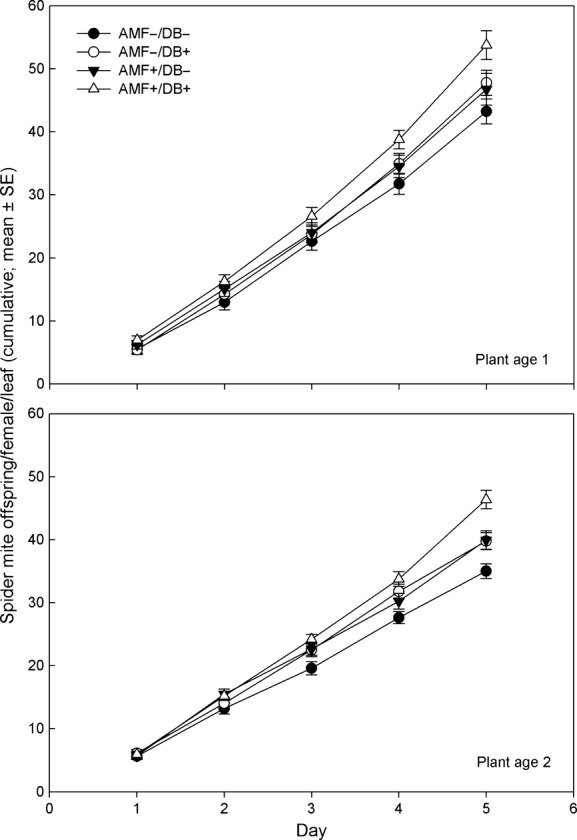
Cumulative number of offspring produced by spider mite females feeding on leaves from bean plants inoculated with arbuscular mycorrhizal fungi (AMF+) and/or diazotrophic bacteria (DB+) or left uninoculated (AMF−, DB−) over 5 days; plant ages were 3–4 weeks (plant age 1) and 5–6 weeks (plant age 2) postinoculation with the microorganisms.

**Figure 3:**
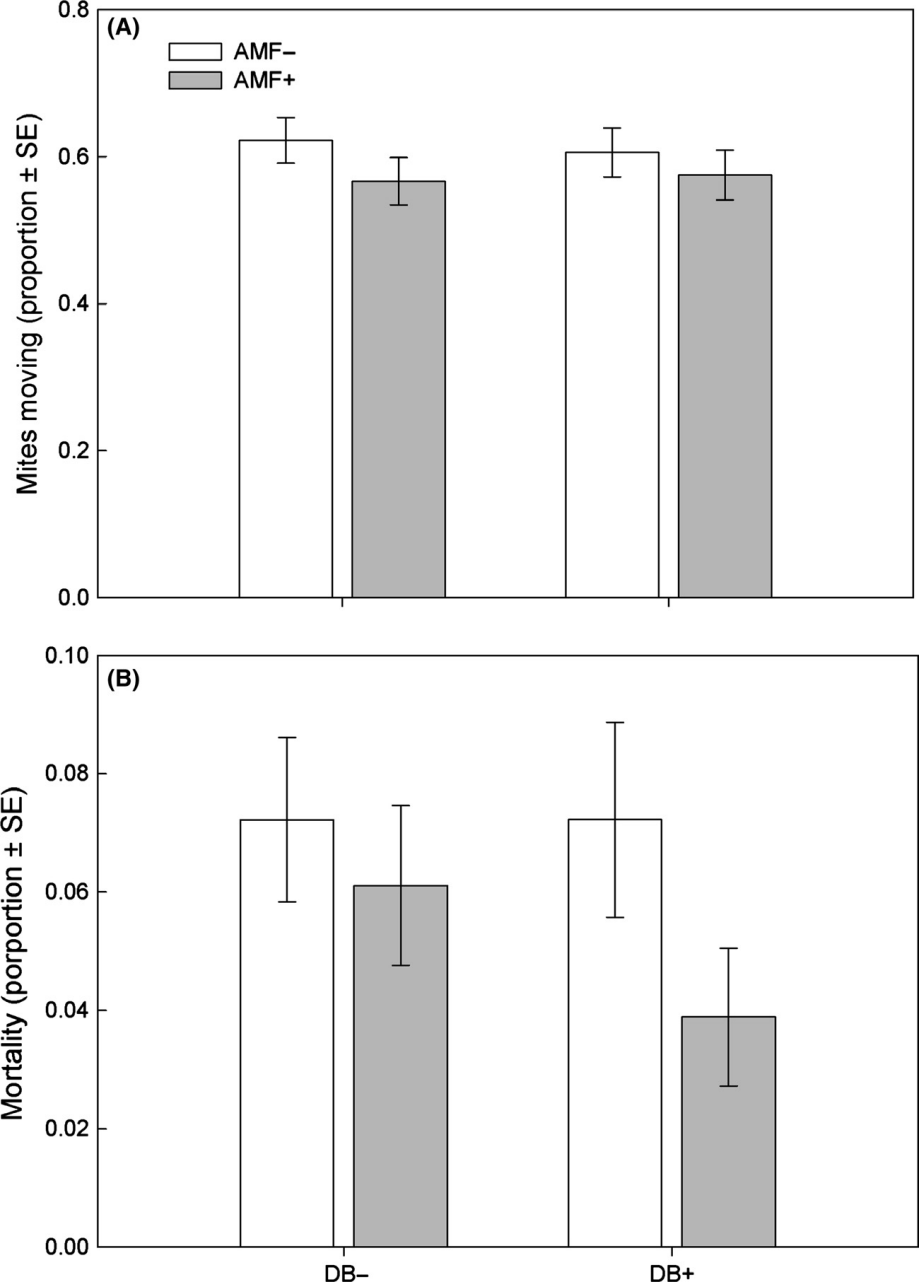
Activity (A) and mortality (B) of spider mite females feeding on leaves from bean plants inoculated with arbuscular mycorrhizal fungi (AMF+) and/or diazotrophic bacteria (DB+) or left uninoculated (AMF−, DB−) over 5 days.

**Figure 4:**
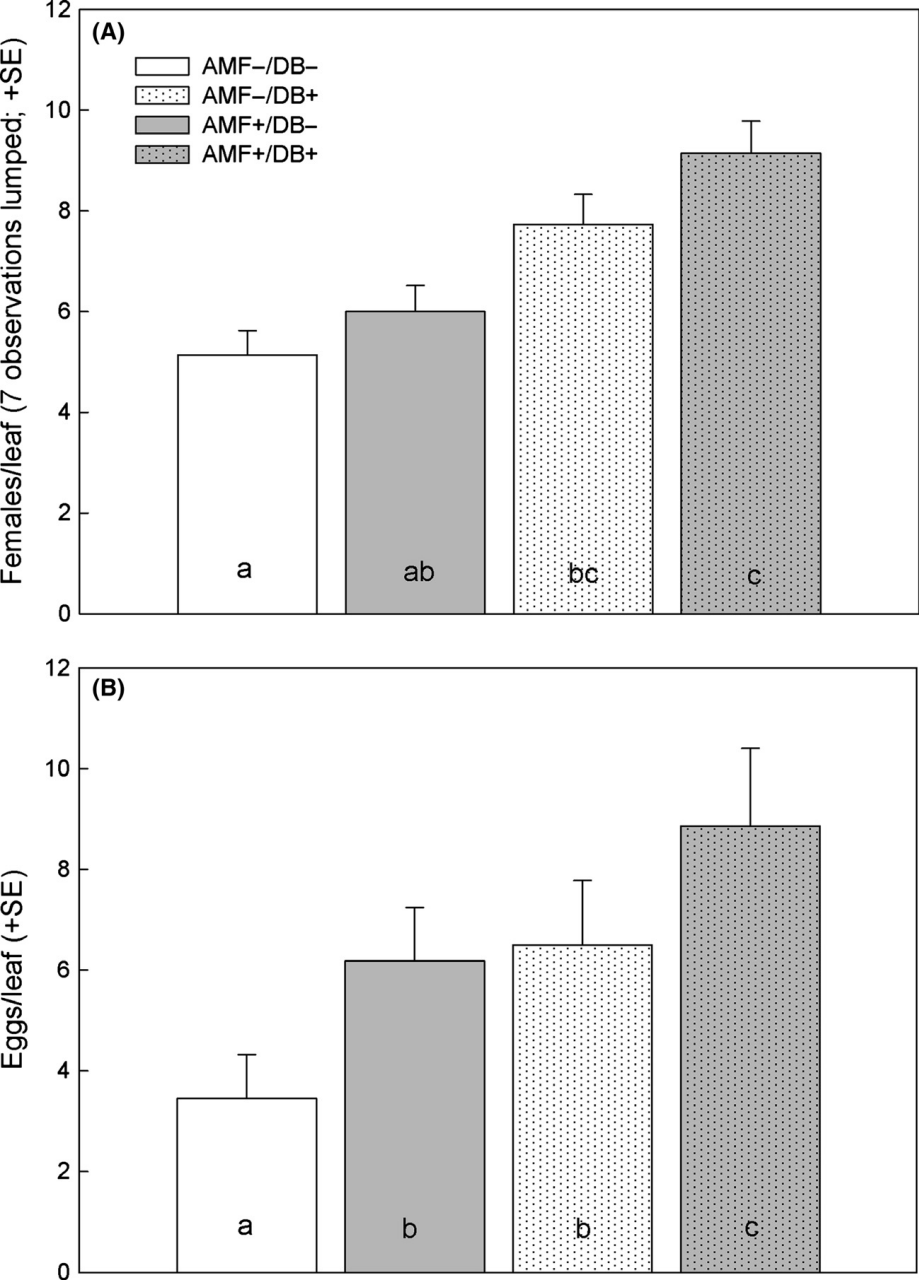
Residence (A; seven observations per choice unit) and oviposition within 24 h (B) of four spider mite females (one raised on AMF−/DB−, AMF+/DB−, AMF−/DB−, and AMF+/DB+ each) given a choice between four interconnected leaves from bean plants inoculated with arbuscular mycorrhizal fungi (AMF+) and/or diazotrophic bacteria (DB+) or left uninoculated (AMF−, DB−). Different lower case letters inside bars indicate significant differences between treatments (Sidak for residence and LSD for oviposition following GEE; *P *<* *0.05).

In the spider mite no-choice experiment, both AMF and DB increased offspring production by the spider mite females, which also produced more offspring on leaves from young than old plants (Fig.2, Table1). Neither AMF nor DB affected spider mite activity, but the mites were overall less active on young than old leaves. None of the two-way interactions affected activity (Fig.3A, Table1). AMF marginally significantly enhanced mite survival, whereas DB, plant age, and the two-way interactions did not have an effect (Fig.3B, Table1). In the spider mite choice experiment, the site preference of the spider mite females was influenced by plant inoculation with AMF and/or DB (Fig.4A; GEE; *Wald ӽ*_3_^2^ = 22.59, *P *<* *0.001) and ranked AMF+/DB+, AMF−/DB+, AMF+/DB−, AMF−/DB− (Sidak; *P *<* *0.05). The oviposition preference (Fig.4B; GEE; *Wald ӽ*_3_^2^ = 39.97, *P *<* *0.001) followed the same ranking pattern, with slight differences in pairwise treatment comparisons (LSD; *P *<* *0.05).

**Table 1:** Results of generalized estimating equations (GEE) for the effects of arbuscular mycorrhizal fungi *G. mosseae* (AMF), diazotrophic bacteria *A. chroococcum* (DB), and plant age (3–4 and 5–6 weeks postinoculation) on oviposition, activity and mortality of two-spotted spider mites *T. urticae* feeding on leaves of common bean *P. vulgaris*

	Oviposition		Activity		Mortality	
Source of variation	*Wald ӽ*_1_^2^	*P*	*Wald ӽ*_1_^2^	*P*	*Wald ӽ*_1_^2^	*P*
AMF	8.400	0.004	2.361	0.124	3.003	0.083
DB	9.470	0.002	0.040	0.841	1.015	0.314
Plant age	99.309	<0.001	18.259	<0.001	0.487	0.485
AMF^*^DB	0.139	0.710	0.207	0.649	0.979	0.322
AMF^*^plant age	0.241	0.624	0.204	0.651	0.007	0.932
DB^*^plant age	0.319	0.572	1.869	0.172	0.082	0.774

AMF, arbuscular mycorrhizal fungi; DB, diazotrophic bacteria.

**Figure 5:**
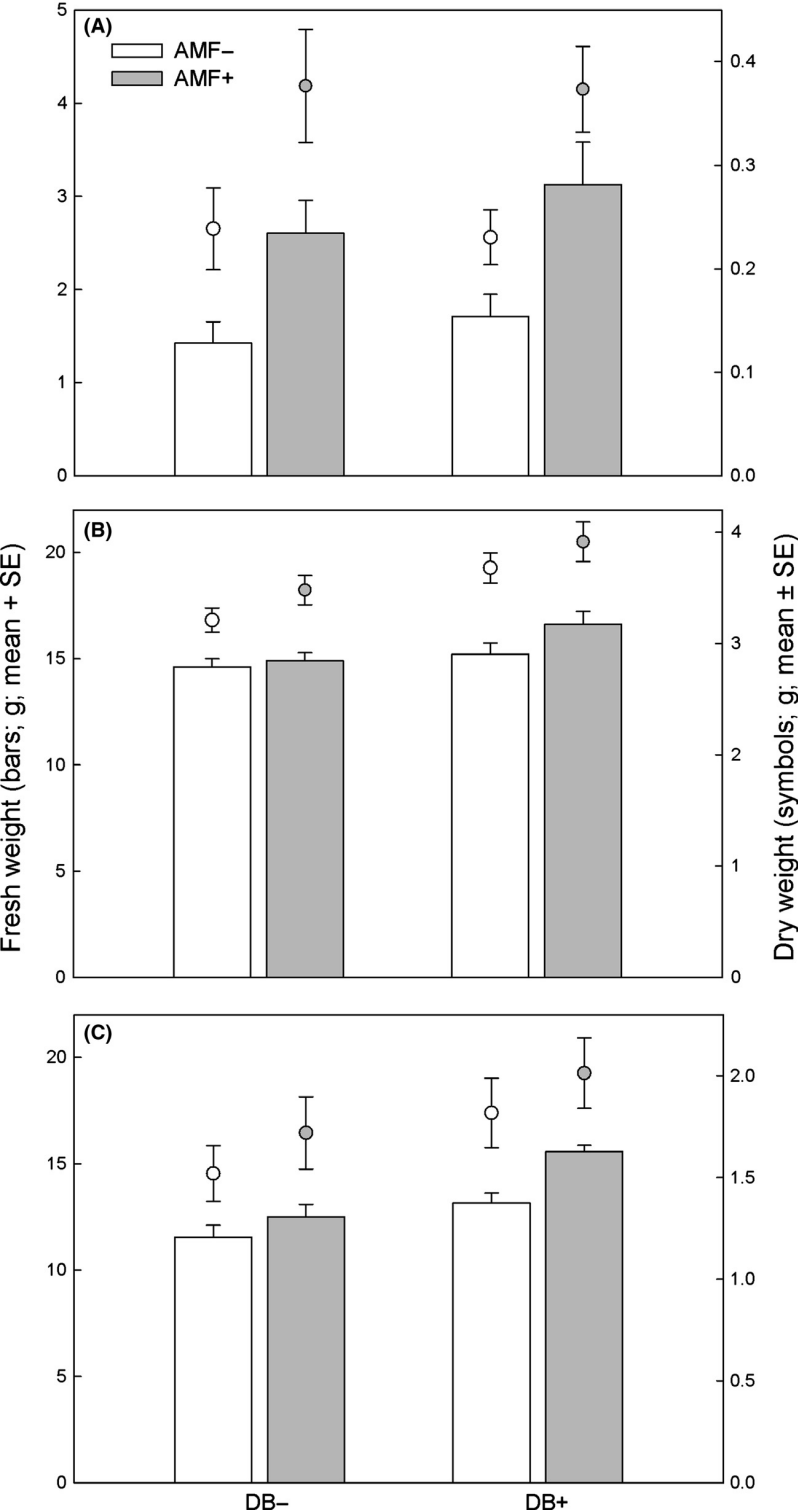
Fresh and dry weight of pods (A), shoots (B), and roots (C) of common bean plants inoculated with arbuscular mycorrhizal fungi (AMF+) and/or diazotrophic bacteria (DB+) or left uninoculated (AMF−, DB−). Data refer to three plants grown together in a pot.

AMF but not DB increased pod weight (Fig.5A, Table2); both AMF and DB increased shoot and root weight (Fig.5B,C, Table2); for neither parameter, the interaction of AMF and DB was significant (Table2).

**Table 2:** Results of generalized estimating equations (GEE) for the effects of arbuscular mycorrhizal fungi *G. mosseae* (AMF), and diazotrophic bacteria *A. chroococcum* (DB), on weight of pods, shoots, and roots of common bean plants *P. vulgaris* (three plants grown together in a pot)

	Pods		Shoots		Roots	
Source of variation	*Wald ӽ*_1_^2^	*P*	*Wald ӽ*_1_^2^	*P*	*Wald ӽ*_1_^2^	*P*
AMF	18.016	<0.001	4.248	0.039	7.946	0.005
DB	1.310	0.252	5.449	0.020	17.924	<0.001
AMF^*^DB	0.181	0.670	0.456	0.500	0.518	0.472

AMF, arbuscular mycorrhizal fungi; DB, diazotrophic bacteria.

**Figure 6:**
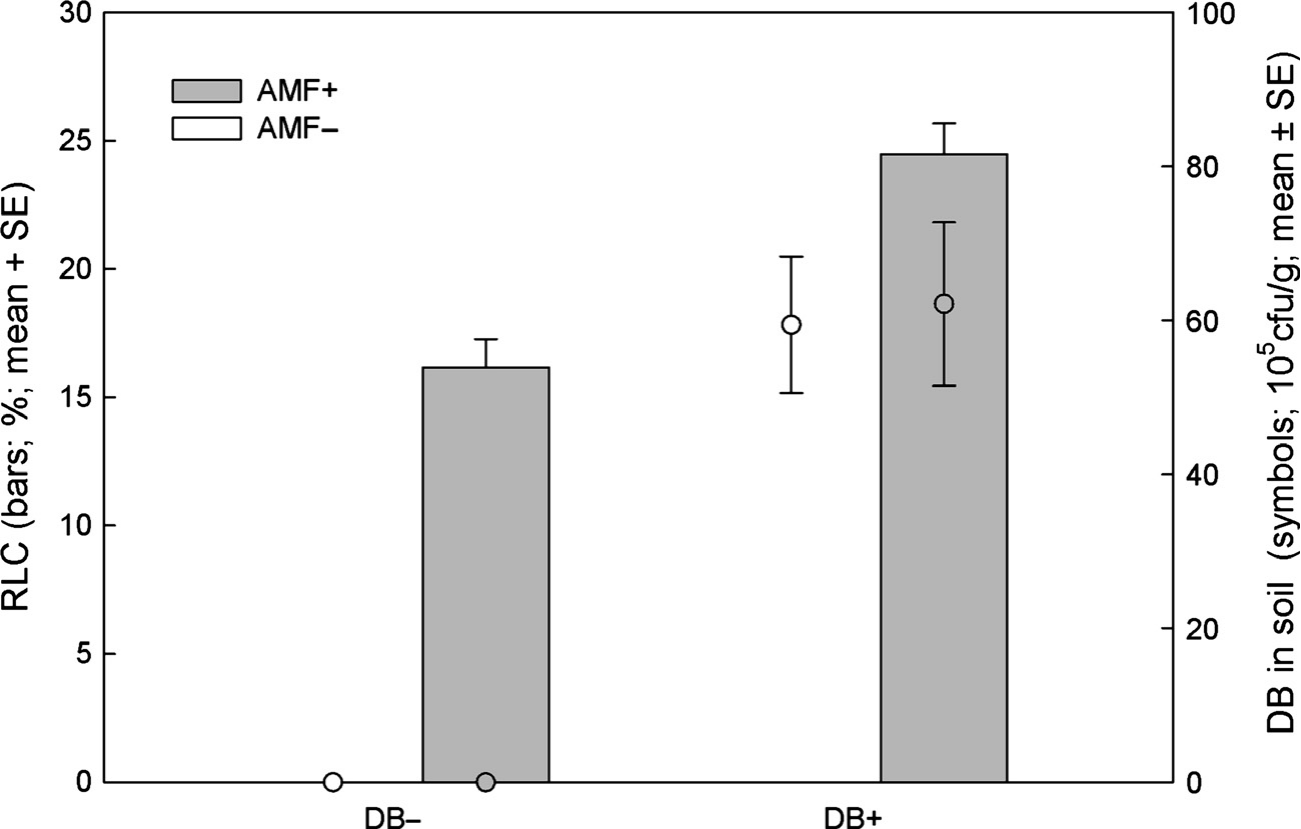
Root length colonized (RLC) by arbuscular mycorrhizal fungi (AMF) in presence and absence of diazotrophic bacteria (DB) and DB density in the soil in presence and absence of AMF.

DB presence increased the RLC of AMF-inoculated plants (*Wald ӽ*_1_^2^ = 27.708, *P *<* *0.001), whereas AMF did not affect the density of DB in the soil of DB-inoculated plants (*Wald ӽ*_1_^2^ = 0.040, *P *=* *0.841) (Fig.6).

## Discussion

The effects of single belowground microorganism species on aboveground plant-associated herbivores are relatively well understood, but virtually nothing is known about the combined effects of functionally distinct belowground microorganisms on the interactions between aboveground plant parts, herbivores, and carnivores. Here, we scrutinized the interrelated effects of two primarily plant-mutualistic belowground microorganisms, a mycorrhizal fungus and a free-living nitrogen-fixing bacterium, on herbivores feeding on aboveground plant parts. In detail, we disentangled the effects of the DB *A. chroococcum* and the AMF *G. mosseae* on host plant choice and life history of the two-spotted spider mite *T. urticae* on common bean plants *P. vulgaris*. In addition, we assessed plant performance as affected by DB and AMF, and AMF performance as affected by DB, which may act as AMF helper bacteria (e.g. Garbaye 1994; Bhowmik and Singh 2004; Behl et al. 2007; Frey-Klett et al. 2007). Both AMF and DB alone increased spider mite reproduction to similar levels and exerted additive effects under co-occurrence. These effects were similarly apparent in host plant choice, that is, the spider mites preferred and performed the best on AMF+/DB+ leaves, followed by AMF−/DB+ and AMF+/DB− leaves, and the worst on AMF−/DB− leaves. DB enhanced root colonization by AMF, whereas the density of DB was unaffected by the presence of AMF. AMF but not DB enhanced reproductive plant growth, that is, seed production. Both DB and AMF increased the biomass of vegetative shoot and root tissue.

Although strongly context dependent (Kiers and Denison 2008; Hoeksema et al. 2010), the most widespread effects of mutualistic soil microorganisms on plants are improvement of the nutritional status and thus promotion of growth and/or enhancement of the defensive system (Smith and Read 1997; Pineda et al. 2010; Friesen et al. 2011). Accordingly, the net effect of a given belowground microorganism on herbivore performance is a trade-off between positive effects, due to improved quality and/or quantity of the host plant, and negative effects, due to strengthened constitutive and/or induced resistance mechanisms (Bennett et al. 2006; Gehring and Bennett 2009; Pineda et al. 2010; Schausberger et al. 2012; Pangesti et al. 2015). In our study, AMF and DB alone increased vegetative plant tissue and, evident from the enhanced life history of the spider mites, the nutritional value of the plant tissue for the herbivores. Together, the two microorganisms had additive effects on both plant growth and spider mite life history and behavior. AMF primarily enhances uptake of P and K by the bean plants of our experimental system (Hoffmann et al. 2009) while DB primarily enhances uptake of N (Mrkovacki and Milic 2001). AMF additionally increased the weight of bean pods, that is, enhanced growth of reproductive tissue, reflecting the strong dependency of seed formation on P availability (e.g. Sterner and Elser 2002).

The provision of additional P and N to plants, up to an upper limit, profoundly affects plant interactions with herbivores, which are strongly P and N limited (Slansky and Rodriguez 1987; Sterner and Elser 2002). Accordingly, variation in P and N content of plant tissue commonly affects host plant choice and life history of herbivores (Coley et al. 2006) including spider mites (Wermelinger et al. 1985, 1991; Hoffmann et al. 2009). For example, N_2_ fixation by rhizobia increases the N content of host plant tissue (Sprent and Sprent 1990), and thereby strongly determines host plant quality to herbivores (Schädler et al. 2007; Chen et al. 2008). However, in addition to primary plant compounds, the concentration and/or composition of secondary metabolites or morphological alterations may crucially determine the outcome of plant–herbivore interactions (Kempel et al. 2009; Katayama et al. 2010). For example, rhizobia can increase the production of alkaloids (Johnson and Bentley 1991) or cyanogenic compounds (Thamer et al. 2011), which are used for direct defense against herbivores. *Azotobacter* sp. may induce morphological changes such as thickening the leaf cell walls and leaf cuticulas (e.g. González et al. 2011), making herbivore attacks more difficult or energy consuming. Rhizobia or *Pseudomonas* colonization may also change volatile emission of plants, affecting the host plant choice of herbivorous beetles (Ballhorn et al. 2013) and third trophic level natural enemies such as parasitoids (Pineda et al. 2013). Therefore, the net effect of mutualistic soil microorganisms on aboveground herbivores depends on the trade-off between the enhancement of nutritional quality versus defensive mechanisms (Pozo and Azcon-Aguilar 2007; Kempel et al. 2010). The *P. vulgaris* variety used in our study is poorly directly but strongly indirectly defended (Hoffmann et al. 2009; Schausberger et al. 2012). Accordingly, we argue that, in our system, the net effects of AMF and DB on the herbivores were largely positive due to enhancement of the nutritional quality of their host plant. Improved uptake of N and P and other elements conceivably enhanced the production and availability of proteins and photosynthates such as sugars in the leaf tissue, all of which are highly important for mite reproduction (Slansky and Rodriguez 1987). However, involvement of third trophic level natural enemies, and enhancement of indirect defense mechanisms, such as the release of herbivore induced plant volatiles (HIPVs), might turn the net effects into negative. For example, Pangesti et al. (2015) observed that *Pseudomonas* sp. changed aboveground volatiles to enhance recruitment of parasitoids to herbivore-infested plants. Similarly, Schausberger et al. (2012) showed that AMF increases the attractiveness of HIPVs, induced by spider mites, to their third trophic level natural enemies, predatory mites. The combined effects of interacting soil mutualists of plants on third trophic level natural enemies have not yet been explored.

Regarding the mutual effects of the microorganisms, our experiments revealed that the DB *A. chroococcum*, which may act as AMF helper bacteria, increased the level of root colonization by the AMF *G. mosseae*. In contrast, AMF did not affect DB occurrence and density. For the nodulating nitrogen fixer *Frankia*, Diem (1996) observed exactly the opposite, that is, positive effects of mycorrhiza on *Frankia* but lacking effects of *Frankia* on mycorrhiza. Mycorrhiza helper bacteria (MHB) are a highly diverse group of bacteria and include *Azotobacter* sp. (Garbaye 1991; O'Connor et al. 2002; Frey-Klett et al. 2007). MHB may enhance formation and establishment of mycorrhiza, the association between plant roots and AMF (Allen 1996; Smith and Read 1997), via diverse mechanisms such as producing substances/nutrients that stimulate AMF and/or modify root exudates and/or stimulate the host to produce substances enhancing mycorrhiza formation (Fitter and Garbaye 1994; Bansal et al. 2002).

Overall, our study provides a key example of the interrelated effects of two primarily plant-mutualistic microorganisms, mycorrhizal fungi and free-living nitrogen-fixing bacteria, on herbivores feeding on aboveground plant parts. It breaks new ground in multitrophic belowground–aboveground research by providing first insights into the implications of plant-mediated belowground fungi–bacteria interactions on fitness of aboveground herbivores.

## Supporting Information

Data S1 . Raw data for oviposition, mortality, activity and host plant choice of the spider mites, vegetative and reproductive plant growth, root colonization by mycorrhiza and density of free-living nitrogen fixing bacteria in the soil.
